# The Sensitivity of Single-Trial Mu-Suppression Detection for Motor Imagery Performance as Compared to Motor Execution and Motor Observation Performance

**DOI:** 10.3389/fnhum.2019.00302

**Published:** 2019-08-30

**Authors:** Kunyu Xu, Yu-Yu Huang, Jeng-Ren Duann

**Affiliations:** ^1^Institute of Cognitive Neuroscience, National Central University, Taoyuan, Taiwan; ^2^Institute for Neural Computation, University of California, San Diego, San Diego, CA, United States

**Keywords:** event-related desynchronization, independent component analysis, motor imagery, mu-suppression, latency

## Abstract

Motor imagery (MI) has been widely used to operate brain-computer interface (BCI) systems for rehabilitation and some life assistive devices. However, the current performance of an MI-based BCI cannot fully meet the needs of its in-field applications. Most of the BCIs utilizing a generalized feature for all participants have been found to greatly hamper the efficacy of the BCI system. Hence, some attempts have made on the exploration of subject-dependent parameters, but it remains challenging to enhance BCI performance as expected. To this end, in this study, we used the independent component analysis (ICA), which has been proved capable of isolating the pure motor-related component from non-motor-related brain processes and artifacts and extracting the common motor-related component across MI, motor execution (ME), and motor observation (MO) conditions. Then, a sliding window approach was used to detect significant mu-suppression from the baseline using the electroencephalographic (EEG) alpha power time course and, thus, the success rate of the mu-suppression detection could be assessed on a single-trial basis. By comparing the success rates using different parameters, we further quantified the extent of the improvement in each motor condition to evaluate the effectiveness of both generalized and individualized parameters. The results showed that in ME condition, the success rate under individualized latency and that under generalized latency was 90.0% and 77.75%, respectively; in MI condition, the success rate was 74.14% for individual latency and 58.47% for generalized latency, and in MO condition, the success rate was 67.89% and 61.26% for individual and generalized latency, respectively. As can be seen, the success rate in each motor condition was significantly improved by utilizing an individualized latency compared to that using the generalized latency. Moreover, the comparison of the individualized window latencies for the mu-suppression detection across different runs of the same participant as well as across different participants showed that the window latency was significantly more consistent in the intra-subject than in the inter-subject settings. As a result, we proposed that individualizing the latency for detecting the mu-suppression feature for each participant might be a promising attempt to improve the MI-based BCI performance.

## Introduction

Motor imagery (MI), rehearsing a motor action without actual movement, has been reported to involve similar brain networks, such as the motor cortex, premotor cortex, supplementary motor area, and prefrontal cortex, as reflected in motor execution (ME; Guillot and Collet, 2005; Lotze and Halsband, 2006; Guillot et al., 2007; Munzert et al., 2009; Collet et al., 2011; Westlake and Nagarajan, 2011; Hétu et al., 2013; Bajaj et al., 2014, 2015; Gallivan and Culham, 2015; Jiang et al., 2015; Ridderinkhof and Brass, 2015; Saiote et al., 2016; O’Shea and Moran, 2017). The potentially functional equivalence between the MI and the ME facilitates the build of the MI-based brain-computer interface (BCI), which provides a communication and control channel between the brain and external devices without overt behaviors (Jeannerod and Decety, 1995). Such noninvasive approach is highly suited for the recovery of the disabled motor system after suffering from a severe stroke (Dunsky et al., 2006, 2008; Turken et al., 2008; Prasad et al., 2010; Inman et al., 2012; Silasi and Murphy, 2014; Bajaj et al., 2015), thus it has been widely reported to use the BCI for stroke rehabilitation and controlling life-assistive devices (Birbaumer et al., 1999; Pfurtscheller et al., 2000; Wolpaw et al., 2002; Neuper et al., 2003; Leuthardt et al., 2004; Müller-Putz et al., 2005; Hochberg et al., 2006; Sharma et al., 2006; Garrison et al., 2010; Ang et al., 2011; Szameitat et al., 2012; Friedrich et al., 2013).

In an MI-based BCI, the mu-suppression feature is one of the most applicable electroencephalographic (EEG) markers (Wolpaw and McFarland, 2004; Miller et al., 2010; Friedrich et al., 2013). Previous studies have indicated that the MI induces the dominant mu-suppression pattern among the alpha (8–13 Hz) frequency band over the contralateral (as well as ipsilateral) sensorimotor area, similar with the performance induced by ME (Pfurtscheller and Neuper, 2001; Munzert et al., 2009; Duann and Chiou, 2016). However, in the literature, although promising results have been achieved by using different algorithms (Guger et al., 2003; Lal et al., 2005; Schröder et al., 2005; Naeem et al., 2006; Blankertz et al., 2008; Arvaneh et al., 2011), EEG features (Pfurtscheller and Neuper, 1997; Lan et al., 2005, 2007; Leeb et al., 2007; Blankertz et al., 2008), or signal processing methods (Bashashati et al., 2007), there remain challenges to apply this technology effectively to the clinical application. One primary problem is that the mu-suppression has manifested in different spatial and temporal properties across different participants during the same task, and may also vary from time to time within the same participant (Asensio-Cubero et al., 2013). Traditional approaches, utilizing a generalized parameter for all participants to control the BCI devices without much attention paid to the observation of each trial or individual performance, have resulted in a discouraging outcome (Wolpaw et al., 2002; Neuper et al., 2003; Ang et al., 2011; Soekadar et al., 2011; Szameitat et al., 2012; Duann and Chiou, 2016). For example, Blankertz et al.’s (2010) study claimed that due to individual difference, almost 15% to 30% of the participants failed to control the BCI system, even though some practice or training was provided before the formal test. Although including more EEG channels that are not related to the ME or MI processes (Lal et al., 2005; Lan et al., 2005, 2007; Schröder et al., 2005; Arvaneh et al., 2011), or recruiting a large population for parametric modeling (Guger et al., 2003) might help increase the accuracy at the individual level, the generalization of such a setting was still not easy to complete, and also for EEG. Therefore, it is highly desirable to explore an effective subject-dependent parameter to measure the mu-suppression feature for enhancing the BCI performance.

Besides the MI condition, the mu-suppression feature has been also reported in motor observation (MO) as well as in ME condition (Pfurtscheller and Neuper, 2001; Neuper et al., 2009; Duann and Chiou, 2016). Such a commonality in the mu-suppression feature made it possible to compare the patterns of the mu-suppression associated with different motor conditions within the same experiment. In addition, it was also made possible by the use of independent component analysis (ICA). The ICA has been proved capable of isolating the pure EEG processes from the artifacts, such as the eye artifacts, muscle activity, environmental noise, and other non-motor-related EEG activities (Jung et al., 2000; Turnip and Kusumandari, 2014), and also capable of extracting the common independent EEG component from different conditions that is thought to be originated from the similar neural substrate (Naeem et al., 2006; Brunner et al., 2007; Rogasch et al., 2014; Winkler et al., 2014). For instance, Onton et al. (2006) used ICA to decomposed single-subject EEG data and to extract independent neural activities for the comparison of equivalent brain activities across participants. Duann and Chiou (2016) also applied ICA to extract the common motor-related independent EEG components for the MI, ME, and MO conditions across participants. Given the merits of the ICA to decompose independent source EEG activities, in the present study, we first attempted to use ICA as a preprocessing step to isolate the common motor-related independent EEG component from the EEG data with three motor conditions combined for each participant. Then, the selected motor-related component was further epoched according to the different motor conditions and computed the time-frequency responses to extract the alpha power time course and detect the mu-suppression feature associated with each of the three motor conditions at a single-trial level. The number of trials in which the mu-suppression feature could be detected was counted to rate the accuracy of the mu-suppression detection for the different motor conditions from the same independent motor-related EEG components. In so doing, we expected to achieve the accuracy of the mu-suppression detection with less influence from the non-motor processes, which might be collected at the same time during the EEG signal acquisition. In the meantime, given the mu-suppression pattern was most distinct in the ME condition as reported in Duann and Chiou (2016), the ME mu-suppression feature could set the highest accuracy standard for the condition of MI and MO to facilitate a self-control comparison scheme. Finally, the same approach was adopted to compare the generalized parameters to the individualized parameters (mainly the window latency in this study) for detecting the mu-suppression feature. Significant improvement could then be achieved by individualizing the window latency for mu-suppression detection.

## Materials and Methods

### Participants

Thirteen healthy, right-handed participants were paid to attend the current study. One of the participants was excluded from further analysis due to bad EEG quality, leaving 12 participants (five females, mean age = 24 years, SD = 3) for further data analysis. All participants had normal or corrected-to-normal vision and no history of neurological or psychiatric disorders. The study protocol was approved by Institutional Review Board (IRB) of China Medical University Hospital, Taichung, Taiwan^1^ (Approval No. DMR100-IRB-221). Written informed consents were obtained from all the participants before the experiment.

### Experimental Paradigm and Data Acquisition

**Figure 1:**
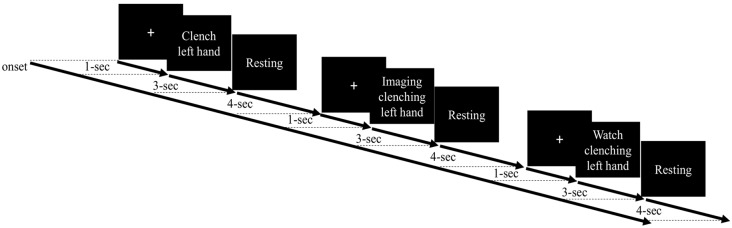
Experimental paradigm with different motor-related tasks, namely, motor execution (ME), motor imagery (MI), and motor observation (MO). Each trial begins with a white cross displayed at the center of a computer for 1 s. Then, a motor cue is presented to indicate which of the three motor tasks follow, and the participant is asked to perform the given action accordingly during the 3-s period. Then, a 4-s resting period is shown before the next trial.

Most MI studies have examined motor cortex function during imagery of dominant hand movement. Moreover, the performance from the non-dominant hand of the healthy participants has been used to set a potential reference for the performance from the paretic hand from the stroke patient and could provide insights into BCI clinical application (e.g., Bauer et al., 2015; Shu et al., 2017). Thus, in this study, we focused on the observation of the non-dominant hand movement and asked the participants to perform the motor tasks using their non-dominant (left) hands. The experimental paradigm contained four EEG runs, and each run consisted of 15 trials of three different motor conditions, namely, ME, MO, and MI, delivered at a random order as shown in Figure 1. Each trial began with a 1-s white cross displayed at the center of a computer screen. Then, a motor cue was displayed to indicate which of the three motor tasks the participants should perform in the following 3 s. For the ME task, the participants were asked to slowly make a fist once using the left (non-dominant) hand during the entire 3-s duration when seeing the motor cue stating “clench left hand.” For the MI condition, the participants were required to kinesthetically imagine clenching the left hand once within the 3-s period, absolutely without actual movement. For the MO trial, the participants needed to watch a 3-s video clip of one real person clenching the left hand slowly in a third-person perspective without making any physical movement. A resting period with a time window of 3–5 s (4 s on average) was appended after the motor cue to make an 8-s period for each trial. Before the formal experiment, the participants were asked to practice to make sure they completely conformed to the requirements of the tasks and held still without any actual movement during the MI and MO conditions. As a result, each EEG run lasted for 120 s, and the whole experiment took about 20 min. The EEG data were recorded using an EEG system (Neuroscan SymAmp2, Computmedics Limited, VIC, Australia) with 64 channels, including two EOG channels, placed according to the International 10–20 system and referenced to the linked mastoids. Hardware band-pass filter (0.1–250 Hz) was applied and the signals were digitized at the sampling rate of 1,000 Hz. The impedance of electrodes was maintained below 5 KΩ.

### Data Processing

The EEGLAB Matlab Toolbox^2^ was used to analyze the EEG data. First, the EEG signals from each participant were screened by the EEGLAB using the Artifact Subspace Reconstruction (ASR)^3^ toolbox to remove bad EEG channels and portions. On average, the ASR removed 7% of the EEG data. The EEG data of one out of the 13 participants had been removed for more than the mean portion [two standard deviations (SDs) higher than the mean] and thus were excluded from the further data analysis. The EEG data were then filtered with a band-pass filter of 1–50 Hz and downsampled to 250 Hz. Then, after removing the two EOG channels, all the data was off-line re-referenced to the common average reference. Afterward, the cleaned EEG data of all runs for each participant were concatenated for the ICA decomposition using an infomax ICA algorithm as implemented in EEGLAB. Finally, the independent motor-related EEG component, which was common to three different motor conditions, was selected from the ICA result of each participant by matching the component topography, the source location, and the time-frequency responses.

### Selecting Independent Mu-Components

**Figure 2:**
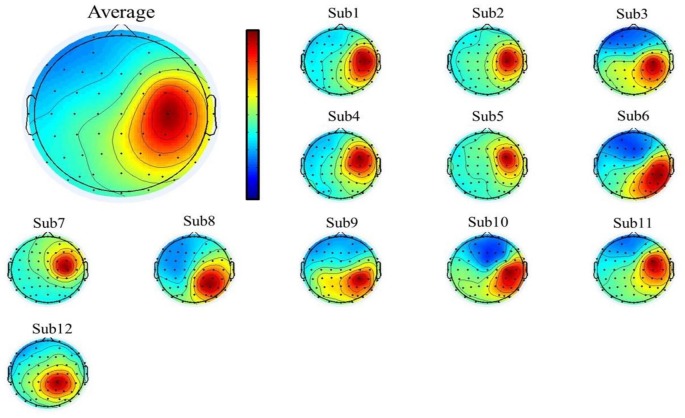
The mean (upper-left corner) and individual topography of the right mu-component of all participants. The topographies show a maximum projection to the C4 channel, corresponding to the right motor-related cortex.

**Figure 3:**
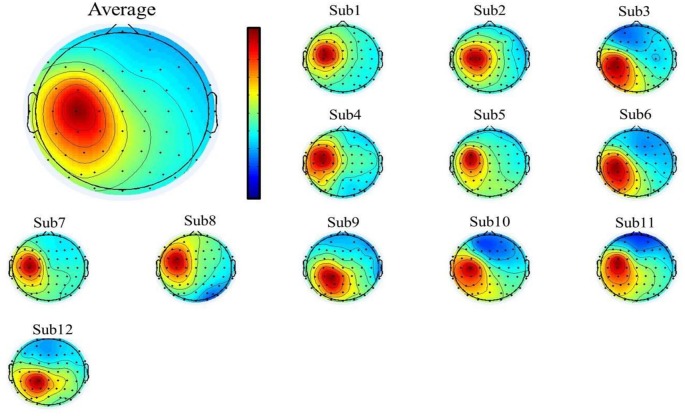
The mean (upper-left corner) and individual topography of the left mu-component of all participants. The topographies show a maximum projection to the C3 channel, corresponding to the left motor-related cortex.

To select the motor-related independent components from all the participants, we first used individual component topography (as shown in Figures 2, 3 below) to identify all centrally located generators, and also with the focus lateralized right to the midline. Moreover, to verify the foci of the components, we localized the sources of all the components using the DIPFIT toolbox and also a standard MNI Boundary Element Model, with the warp montage function to co-register the electrode locations with the head model, followed by coarse and fine fitting (Delorme and Makeig, 2004). In addition, we also performed the time-frequency analysis (using the EEGLAB default setting, a three-cycle Morlet wavelet with a Hanning-tapered window) on these components to identify the mu-components from each of the 12 participants. Given all the participants were right-handed, when they were asked to perform the motor action using the left hand, it was more likely that the contralateral (right) mu-component would dominate the task performance; however, the ipsilateral (left) mu suppression due to hand dominance could be also expected to be symmetrically found in a separate independent component (as shown in below Figure 3).

### Evaluating the Success Rate of the Mu-Suppression Detection

After the mu-components were identified, the continuous component activities were epoched from −0.5 s before and 3 s after the onset of the motor cue, and all the epochs were categorized into three trial types (ME, MI and MO). Note that the feature we selected for further comparison was based on the average group time-frequency results that displayed the most consistent time-frequency feature across participants (and eventually the mu-suppression feature within the alpha frequency band). Then, the alpha power time course was extracted by averaging the time-frequency results within the selected frequency range of 8–13 Hz. Given that the frequency band (8–13 Hz) for the mu-suppression features was quite robust across different participants, especially in the ME task (Duann and Chiou, 2016), we thus adopted this feature for the mu-suppression detection. In the meantime, because this feature was more pronounced in the ME condition, it thus set a gold standard to compare the results of the other two motor condition to that of the ME condition. Hence, we here used the fixed alpha frequency band (8–13 Hz) for mu-suppression feature extraction.

The success of detecting the mu-suppression pattern in a single motor trial was determined by if we could successfully find a time window in which the alpha power was significantly lower than the alpha power in the baseline window. First, we computed the time-frequency decomposition using a three-cycle Morlet wavelet for each epoch. The number of cycles in the wavelets used for higher frequencies would continue to expand to half of the number of cycles at the highest frequency (the default setting in the EEGLAB). Each EEG epoch consisted of a 200-ms window with 90% overlap from the previous time window to result in the time-frequency response for each trial at high temporal resolution (~20 ms). Subsequently, we applied a sliding window approach with no overlap to compare the alpha power within the moving window after the motor cue onset to the alpha power of the pre-motor cue baseline along the alpha power time course. The size of the baseline window was the same as the moving window for further comparison. If the alpha power in any of the moving windows was significantly lower than the alpha power in the baseline window using a paired *t*-test (at the significance level of *p* < 0.05 with Bonferroni multiple comparison corrections), this trial was then labeled as a successful trial with mu-suppression. To determine the window size of the moving window (as well as the baseline) for effectively comparing the alpha power, we then compared the success rate from different window sizes varying from 150 to 500 ms (i.e., 150 ms, 200 ms, 260 ms, 320 ms, 380 ms, 440 ms, and 500 ms). For each window size setting, the moving window was sliding through the entire alpha power time course for each trial to determine if the mu-suppression pattern existed in the current trial. Then, the success rate was further summarized for each participant by counting how many trials out of the total trials exhibiting a successful mu-suppression detection. After selecting the window size setting for each participant and condition, we further compared the success rate of the mu-suppression feature detection for each participant and motor condition by using a non-parametric Kruskal–Wallis test. The *H*-value with degrees of freedom and the corresponding *p*-value was reported in the results. In addition, Dunn’s pairwise tests with Bonferroni correction at an alpha value of 0.05 were also performed to validate the pairwise difference.

### Selecting the Window Size and the Window Latency

To improve the sensitivity of the mu-suppression detection on each motor condition, we selected a window size to detect the mu-suppression features at the single-trial level for all participants mainly according to the following three criteria: first, the window size should deliver the best success rate of mu-suppression detection; second, considering the real-time characteristic of the BCI operation, the window size should be relatively short to expedite the feature detection process. Finally, the window size should contain enough sample points to provide sufficient frequency resolution to result in multiple frequency bins within the 8–13 Hz range for computing the mean alpha power.

In addition to selecting the window size, we further used the same sliding-window approach with the 150-ms window size to determine the window latency that delivered the best success rate of detecting the mu-suppression features for each motor condition and participant. Besides, to evaluate the efficacy of such individualized parameter, we further compared the success rate using the individualized latency with the success rate utilizing a generalized latency. Here, the generalized window latency of 635 ms was determined by the grand average of the time-frequency plot obtained from all the motor conditions and all the participants.

## Results

### The Mu-Components for the Motor-Related Cortex

A single ICA decomposition was applied to individual data with all the three motor conditions combined, thus the common right-mu and left-mu-components for all motor conditions could be identified for each participant as shown in Figures 2, 3, respectively. Given that in our study, the participants were required to complete the task using the left (non-dominant) hand, we thus focused on the analysis and interpretation of the contralateral (right mu) components across participants and then compared the observed results with that of the ipsilateral components.

### The Success Rate Under Different Window Sizes

**Figure 4:**
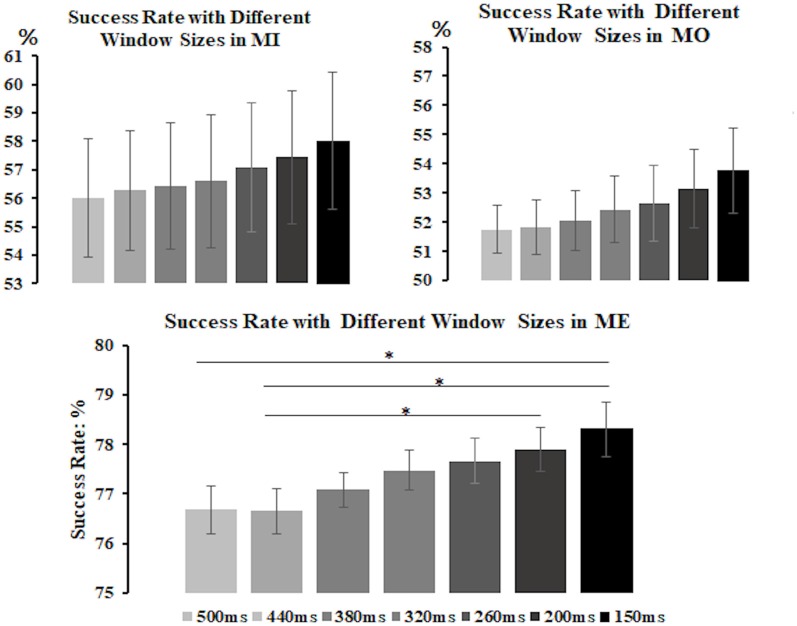
The average success rate (y-axis) with different window sizes (x-axis) in the MI, MO and ME conditions across participants. The error bars represent the 95% Confidence Interval (CI). The formula to calculate 95% CI is Mean ± 1.96 × Standard Error. The results show that the 150-ms window size tends to deliver the best average success rate among all in the MI, as well as the ME and MO conditions, thus the 150-ms window is preferred to be the suitable window for computing the success rate of detecting the mu-suppression feature for each motor condition and participant. *p < 0.05.

All three motor conditions could induce a significant mu-suppression response in the alpha frequency band after the motor task performance as compared to the baseline (see Supplementary Material Figure S1). To select the window size for further analysis, we derived the alpha power from the contralateral (right-mu) components and separately calculated the success rate of detecting the mu-suppression features by using different window sizes, varying from 150 ms to 500 ms, for each motor condition and participant, as displayed in Figure 4. The results from a non-parametric Kruskal–Wallis test showed that there was no significant difference in success rate across different window sizes in the MI (*H*_(6)_ = 3.116, *p* = 0.794) and the MO condition (*H*_(6)_ = 4.888, *p* = 0.558) condition. However, in the ME condition, the window size did exhibit a significant effect on the success rate (*H*_(6)_ = 28.904, *p* < 0.001). The *post hoc* analysis with Bonferroni correction further indicated that the success rate (mean ± SD) under the window size of 150 ms (78.31 ± 1.68%) was significantly higher than that under the window size of 500 ms (*p* = 0.005) and 440 ms (*p* = 0.001), showing the tendency toward delivering highest success rate among all window size settings. Although in the MI and MO conditions, no significant difference in success rate was observed across various window size settings, the window size of 150 ms in the MI (58.02 ± 7.23%) and the MO (53.77 ± 4.35%) conditions still tended to deliver a higher success rate than other window size settings. The consistent pattern was also presented when we derived the alpha power from the ipsilateral components, showing that the window size of 150 ms delivered the best accuracy compared to the other window settings. Therefore, based on the statistical comparison as well as the selection criteria mentioned above, the window size of 150 ms was eventually selected to compute the success rate of the mu-suppression detection for each motor condition and participant.

**Figure 5:**
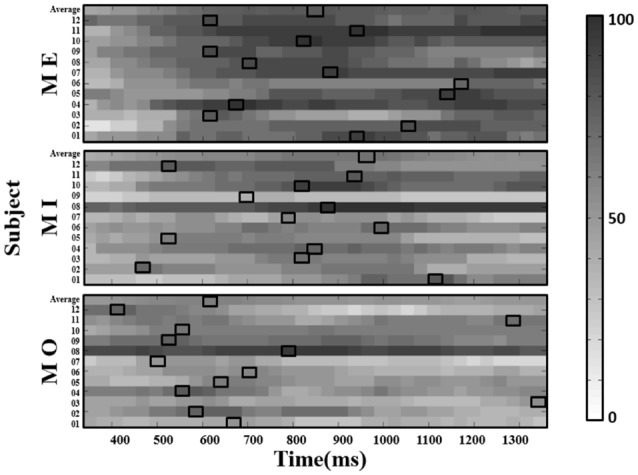
The window-by-window plots of the success rate (in percentage, see color bar) for all trials concatenated from four runs in the ME, MI and MO conditions, using the alpha power derived from the contralateral (right) mu-components. The x-axis indicates the time in ms. The various gray-level blocks of each row indicate the success rate derived using the 150-ms sliding window at the latency indicated by the x-axis along the alpha power time course from the motor cue onset. The best success rate in each participant and average across participants (top row) are marked with the empty black boxes. The results show that the latency delivering the best success rate in the MI condition, as well as the ME and MO conditions, is found to be dramatically different across participants.

With the selected window size of 150 ms, we depicted the window-by-window plot to see whether and how the success rate varies across different window latencies along the alpha power time course for each motor condition and participant (see Figure 5). In the figure, each panel represents a motor condition; each row represents the success rate derived from one participant (the average across participants shown at the top of each panel). Each small-block indicates each success rate derived using the 150-ms sliding window at the latency indicated by the x-axis along the alpha power time course from the motor cue onset. The gray-level blocks indicate the success rate, the darker, the higher success rate is. The empty black box highlights the best success rate for each motor condition from each participant. As shown in Figure 5, the success rate largely varies across different window latencies in each participant, and the latency delivering the best success rate in the MI condition also widely differ from those in the ME and MO conditions across participants. Individual performance of the success rate in each of the three motor conditions was also summarized in Table 1. As shown in Table 1, all three motor conditions exhibit a large SD in the success rate for each participant. The highest SD can go as high as 17.0% in the MI, 17.0% in the MO, and 20.6% in the ME condition.

**Table 1:** Individual performance of the success rate in motor imagery (MI), motor execution (ME) and motor observation (MO) conditions: mean and standard deviation (SD).

	MI		ME		MO	
Subject	*Mean*	*SD*	*Mean*	*SD*	*Mean*	*SD*
1	51.1	17.0	71.8	12.8	38.1	5.2
2	55.2	9.4	67.0	20.6	55.6	13.4
3	50.6	10.6	56.6	15.4	43.3	5.7
4	64.4	7.8	83.6	15.1	58.5	9.6
5	50.7	11.3	72.3	14.6	49.5	8.1
6	59.7	7.2	60.6	9.9	44.8	7.4
7	43.3	10.2	79.5	16.0	34.9	10.0
8	89.0	9.3	69.8	14.0	88.8	4.6
9	31.0	4.5	71.2	10.6	67.5	5.4
10	71.9	15.7	78.3	14.1	60.8	5.6
11	64.1	16.6	86.8	17.4	59.0	7.7
12	65.3	11.0	79.0	6.7	44.4	16.7
Average	58.0	10.9	73.0	13.9	53.8	8.3

**Figure 6:**
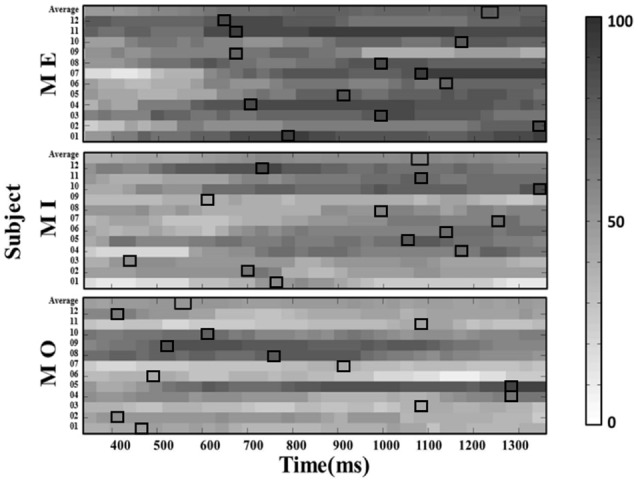
The window-by-window plots of the success rate in all motor conditions using the alpha power derived from the ipsilateral (left) mu-components. The x-axis indicates the time in ms. The various gray-level blocks of each row indicate the success rate derived using the 150-ms sliding window at the latency indicated by the x-axis along the alpha power time course from the motor cue onset. The best success rate in each participant and average across participants (top row) are marked with the empty black boxes. The results show that the latency delivering the best success rate in all motor conditions is also dramatically different across participants, as observed from the contralateral mu-component.

Further, we computed the average success rate across the 150-ms windows at all latencies along the alpha power time course for each motor condition and participant. The results from a Kruskal–Wallis test showed that the average success rate also significantly varied from participant to participant in all three motor conditions (MI: *H*_(11)_ = 244.042, *p* < 0.001; ME: *H*_(11)_ = 134.932, *p* < 0.001; MO: *H*_(11)_ = 272.915, *p* < 0.001). On the other hand, the similar individual-difference pattern was also observed when we computed the success rate of detecting the mu-suppression from the ipsilateral components (as shown in Figure 6).

Considering that there was a large inter-subject variation as displayed, we further examined the potential individualized parameter for BCI improvement as follows.

### The Mu-Suppression Detection Using Different Latency Settings

**Figure 7:**
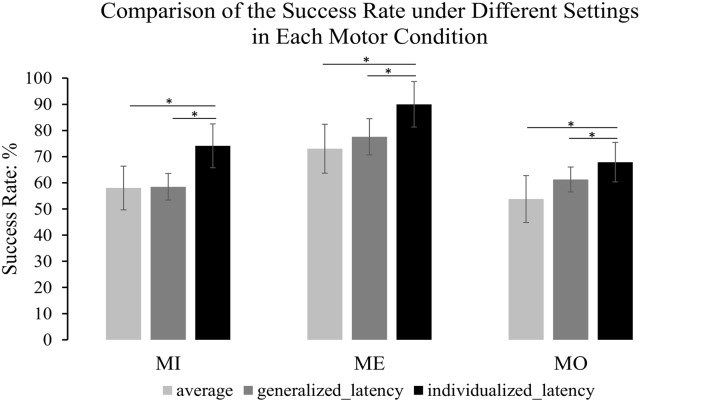
The comparison of the success rate under different settings. The light gray refers to the average success rate across the 150-ms windows at all latencies along the alpha power time course; the dark gray refers to the success rate under the generalized latency (635 ms after the motor cue) that was determined by the maximum mu-suppression in the grand average time-frequency plot from all the motor conditions and all the participants; the dark black represents the success rate under the individualized latency reflecting the best performance. The error bars refer to 95% CI. The formula to calculate 95% CI is Mean ± 1.96 × Standard Error. The results illustrate that the success rate was significantly improved by an individualized latency compared to that under a generalized latency and also the average success rate in each motor condition. *p < 0.05.

We compared the success rate under the individualized latency reflecting the best performance of each participant, the success rate under the generalized latency at 635 ms after the motor cue onset that was determined by the maximum mu-suppression in the grand average time-frequency plot from all motor conditions and all participants, and also the average success rate across the 150-ms windows at all latencies along the alpha power time course derived from the contralateral mu-components. The results from pairwise comparison tests indicated that using the individualized latency significantly improved the success rate compared to using a generalized latency in all the three motor conditions (MI: 74.14% vs. 58.47%, *t*_(11)_ = 4.766, *p* = 0.001; ME: 90.0% vs. 77.57%, *t*_(11)_ = 3.913, *p* = 0.002; MO: 67.89% vs. 61.26%, *t*_(11)_ = 3.988, *p* = 0.002), and also the average success rate in each motor condition (MI: 74.14% vs. 58.02%, *t*_(11)_ = 9.335, *p* < 0.001; ME: 90% vs. 73.04%, *t*_(11)_ = 12.604, *p* < 0.001; MO: 67.89% to 53.77%, *t*_(11)_ = 7.080, *p* < 0.001). The comparison of the success rate under each setting was clearly illustrated in Figure 7 to present the extent of the improvement in each motor condition. Meanwhile, we also separately computed the success rate for each of the four runs within the same participant to assess the stability of the latency across runs. The results with a Kruskal–Wallis test indicated that there was no significant difference across runs with the selected latency to achieve the best success rate in individuals for each of the three motor conditions (MI: *H*_(3)_ = 1.172, *p* = 0.760; MO: *H*_(3)_ = 2.209, *p* = 0.530; ME: *H*_(3)_ = 4.876, *p* = 0.181). As can be seen, the intra-subject variation was less pronounced than the inter-subject variation, thus such a statistical result further supported the approach to combine the four-run data to ensure the statistical power with a large trial number for each of the motor conditions.

Further, to examine whether the individualized latency could induce the similar improvement from the ipsilateral mu-component, we calculated and compared the success rate under the individualized and generalized latency by using the alpha power derived from the ipsilateral mu-components. The results from pairwise comparison tests showed that a significant improvement was also achieved by using the individualized latency in all the three motor conditions compared to a generalized latency (MI: 69.50% vs. 50.14%, *t*_(11)_ = 5.630, *p* < 0.001; ME: 85.18% vs. 69.28%, *t*_(11)_ = 5.678, *p* < 0.001; MO: 63.50% vs. 50.58%, *t*_(11)_ = 3.464, *p* = 0.005) and also the average success rate (MI: 69.50% vs. 52.82%, *t*_(11)_ = 9.364, *p* < 0.001; ME: 85.18% vs. 68.97%, *t*_(11)_ = 10.03, *p* < 0.001; MO: 63.50% vs. 48.42%, *t*_(11)_ = 5.763, *p* < 0.001). We also found that the grand latency (mean ± SD) to induce the best performance of all the motor conditions tend to be longer in the ipsilateral components compared to that in the contralateral components across participants (MI: 950.96 ± 290.60 ms vs. 810.79 ± 190.10 ms; ME: 931.33 ± 233.96 ms vs. 850.21 ± 207.06 ms; MO: 781.33 ± 340.86 ms vs. 656.08 ± 225.09 ms).

## Discussion

In the present study, by using the approach combining the ICA with a sliding window for detecting the mu-suppression at the single-trial level, we reveal a significant variation of the success rate of detecting the mu-suppression features within and across individuals in the MI condition, as well as in the ME and MO conditions. The success rate in the MI could go as high as 100% for some participants; on the contrary, it could also go as low as 20% for others. Moreover, the results indicated that the success rate from each participant could largely vary across different window latencies (see Figures 5, 6). Therefore, the present results may suggest that the traditional way to find the mu-suppression feature using a generalized parameter set, such as the generalized latency, for all participants to compare the alpha power with the baseline power, might be problematic. Given so, simply evaluating the training performance for an MI-based BCI without justifying the success rate of the single-trial mu-suppression detection might not be sufficient to provide a fair judgment on the training efficacy. It would be difficult to conclude of a failure in the training performance is mainly due to an inappropriate training protocol or caused by the low success rate in detecting the mu-suppression at the operation level. Therefore, the proposed method by recording the success rate of mu-suppression detection in a trial-by-trial fashion might help to reflect the true problem associated with the MI-based BCI training system.

For the detection of mu-suppression features in different motor conditions, we found that the shorter window length setting (150 ms) was preferred to use than the longer window size settings for computing the success rate of detecting the mu-suppression feature for each motor condition and participant. An ME task has been found to mainly activate brain activation in areas associated with ME, such as primary motor cortex, premotor area, the supplementary motor area and even the primary sensory cortex (e.g., Lotze and Halsband, 2006; Hanakawa et al., 2008; Munzert et al., 2009; Collet et al., 2011; Bajaj et al., 2014, 2015; Gallivan and Culham, 2015; Jiang et al., 2015; O’Shea and Moran, 2017), thus the ME may involve the loop interaction between those brain areas from the initiation to the end of a physical movement. As a result, it should induce a long lingering mu-suppression response, as reflected in a deepest and longest mu suppression pattern. On the other hand, both the MI and MO conditions might not involve much of the interaction among those motor-related areas, thus the characteristics of the mu-suppression patterns in both the MI and MO conditions, as compared to the ME conditions, seems to be less stable with much shallower suppression amplitude, much shorter suppression period and large fluctuations (see Supplementary Material Figure S1). The shorter window length thus might be more favorable for the paired *t*-test (here we used to determine the significance of the power difference between the sliding window and the baseline window) to survive and then to detect the potential mu-suppression patterns in MI and MO conditions. Following these reasons, the shorter window length setting, as compared to the longer window length settings, did provide better average accuracy across all three motor conditions in this study (as shown in Figure 4).

Further, the current findings (see in Figure 7) clearly illustrated that if an individualized window latency was selected to derive the mu-suppression feature from the contralateral components, the success rate could be significantly improved than if the parameter was averaged across the entire time window after the motor cue onset (ME, from 73.04% to 90%; MI, from 58.02% to 74.14%; MO, 53.77% to 67.89%), or if a generalized window latency at 635 ms after the motor cue was used for all participants (ME, from 77.57% to 90%; MI, from 58.47% to 74.14%; MO, 61.26% to 67.89%). To be noted, although we do not implement a “NoGo” condition to contrast with the results of the motor conditions in the experiment, the performance in the ME can play the role of benchmarking the performance of the mu-suppression detection in the MI and MO conditions without actual physical movements. In this study, the common mu-components, which provide the maximally isolated motor-related EEG activity with less contamination from other brain or non-brain processes, are selected from each participant for further comparison using a joint ICA decomposition, thus the result of the ME condition can set an upper bound of the success rate for the other motor conditions. That is, the success rate in the MI condition can by no means reach the high standard set by the ME condition, given a much less pronounced mu-suppression feature associated with MI. Besides, although the similar improvement with the individualized latency can also be achieved in all the motor conditions by using the ipsilateral component, the latency to induce the best performance in all motor conditions seems to be shorter in the contralateral side compared to that in the ipsilateral side. Taken together, these results at least drop a hint to us that the individualized latency in the current study indeed improves the success rate and extracting the mu-suppression feature from the contralateral size might be the optimal option for the on-line detection.

To date, increasing interests have been raised in finding an effective subject-dependent approach to improve BCI performance. Previous studies have shown that training BCI systems on subject-specific EEG data before the real-time BCI application can somehow improve BCI performance. For example, Guger et al. (2003) found that high accuracy could be achieved if the MI-based BCI system with a large population was trained on individual EEG data for a subsequent run with specific tasks. However, as introduced above, even if some training was provided before the formal test, there were still almost 15%–30% of users who failed to use a BCI with sufficient level of control (e.g., Guger et al., 2003; Blankertz et al., 2010). Moreover, the generalization of such a large population was not easy to complete in most cases. Therefore, based on the previous findings as well as the current results, we propose that the utilization of individualized EEG features, such as individualized latency, rather than generalized characteristics may be a promising solution to improve the success rate of detecting mu-suppression in an MI-based BCI application.

One may concern that the different performance across conditions is partially due to the experimental design. For example, Annett (1995) found that the variety of MI performance led to individual differences in MI-based EEG changes. Specifically, the subject might, for example, perform the MI task from the first-person perspective (i.e., kinesthetic imagery) or the third-person perspective (visual imagery; Curran and Stokes, 2003). To observe how the EEG pattern changes with different types of MI, Neuper et al. (2005) designed an EEG study and claimed that only kinesthetic experiences instead of visual representations of actions induced the MI-based EEG change. However, such a difference (kinesthetic vs. visual imagery) should not account for the difference in the mu-suppression responses induced by different motor tasks in this study, because all participants are asked to perform both the kinesthetic (MI) and the visual imagery (MO) in the same experiment run, and all participants have reported that they can differentiate these two different motor tasks well. Moreover, some previous BCI studies reported the around event-related synchronization (ERS) effect during motor tasks. The absence of the ERS effect in the current study may be because it only involves one slow hand grasp action in each of the motor conditions. Consequently, we did not see the ERS effect in the time-frequency results given the epoch length we used in the data analysis. That is also the reason why we eventually determine the data analysis parameter using the average group time-frequency responses in this study. Given that in this study we mainly focus on the fixed frequency band to measure mu-suppression features, future work is still needed to investigate whether individual frequency band will influence the BCI performance and also the efficacy of individualized parameters as suggested.

Taken together, to achieve the expected success rate of the mu-suppression feature detection in an MI-BCI performance, conducting a short ME/MI run with few trials before the BCI training to find the individualized parameters may be helpful and such parameters will be then applied for this specific subject across the whole run. Even if the detection performance in the BCI application is still not as good as expected, we would like to propose that the limited success rate of mu-suppression detection should not hinder the delivery of the MI-based BCI applications to those who are targeted to use for their rehabilitation training. First, the performance of operating the BCI system of these targeted users may be gradually improved by practicing. More importantly, the effects of the clinical rehabilitation training in facilitating brain activity using the MI-based BCI apparatus might have already been visible even when the performance accuracy is still limited. However, the parameters might need to be optimal again for the next BCI run, especially for those stroke patients, since their mu-suppression features might change due to the brain reorganization through some effective BCI rehabilitation training.

## Ethics Statement

This study was carried out in accordance with the recommendations of the Social and Behavioral Research Ethical Principles and Regulations of National Taiwan University with written informed consent from all the participants. The protocol was approved by the Research Ethics Committee of National Taiwan University.

## Author Contributions

The experiment was designed by J-RD. Y-YH performed data collection and analysis. KX wrote the draft of the article. J-RD and KX were responsible for modifying the article. All of the authors made their own contributions to the final article and all have agreed to the submission of this version.

## Conflict of Interest Statement

The authors declare that the research was conducted in the absence of any commercial or financial relationships that could be construed as a potential conflict of interest.

## Footnotes

^1^http://www.cmuh.cmu.edu.tw

^2^http://sccn.ucsd.edu/eeglab/index.php

^3^https://sccn.ucsd.edu/wiki/EEGLAB_Extensions
