# Supplementary material for: The Sensitivity of Single-Trial Mu-Suppression Detection for Motor Imagery Performance as Compared to Motor Execution and Motor Observation Performance
Source: Front Hum Neurosci. 2019 Aug 30;13:302. doi: 10.3389/fnhum.2019.00302 (PMC6728805; doi:10.3389/fnhum.2019.00302)
Supplement: Supplementary file 1 [file Data_Sheet_1.docx]

**Supplementary Material A**


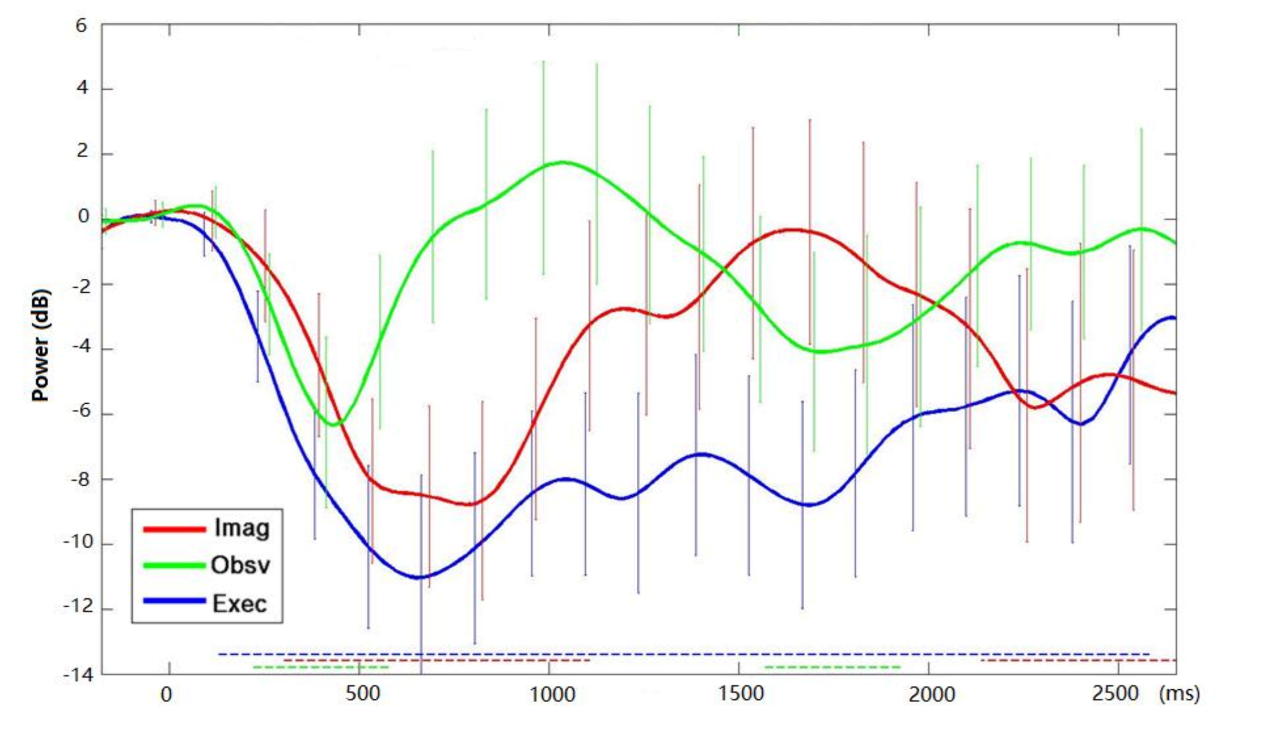


**Fig. S1.** The grand mean conditional alpha ERD time course derived from each of the three motor conditions. The blue line shows the mean alpha ERD time course of ME condition, the red line shows the MI condition and the green line shows the MO condition. The error bars show the standard deviation of the time course at each time point across all trials and participants. The dot lines at the bottom give the time windows with significant alpha suppression from the base line (at the significance level of 0.05), blue for ME, red for MI and green for MO condition. The results show that ME induces a longest and deepest mu suppression pattern, while the characteristics of the mu-suppression patterns in both the MI and MO conditions seems to be less stable with much shallower suppression amplitude, much shorter suppression period and large fluctuations, compared to the ME conditions.
